# A Three-dimensional Floating Air Cathode with Dual Oxygen Supplies for Energy-efficient Production of Hydrogen Peroxide

**DOI:** 10.1038/s41598-018-37919-3

**Published:** 2019-02-12

**Authors:** Haichuan Zhang, Yingjie Li, Hao Zhang, Guanghe Li, Fang Zhang

**Affiliations:** 1 School of Environment and State Key Joint Laboratory of Environment Simulation and Pollution Control, Tsinghua University, Beijing, 100084 China; 2 Key Laboratory for Solid Waste Management and Environment Safety (Tsinghua University), Ministry of Education of China, Tsinghua University, Beijing, 100084 China; 3 Department of Materials Science and Engineering, College of Engineering, Peking University, Beijing, 100871 P. R. China

## Abstract

The *in situ* and cleaner electrochemical production of hydrogen peroxide (H_2_O_2_) through two-electron oxygen reduction reaction has drawn increasing attentions in environmental applications as an alterantive to traditional anthraquinone process. Air cathodes avoid the need of aeration, but face the challenges of declined performance during scale-up due to non-uniform water infiltration or even water leakage, which is resulted from changing water pressures and immature cathode fabrication at a large scale. To address these challenges, a three-dimensional (3-D) floating air cathode (FAC) was built around the commercial sponge, by coating with carbon black/poly(tetrafluoroethylene) using a simple dipping-drying method. The FAC floated on the water-air interface without extensive water-proof measures, and could utilize oxygen both from passive diffusion and anodic oxygen evolution to produce H_2_O_2_. The FAC with six times of dipping treatment produced a maximum H_2_O_2_ concentration of 177.9 ± 26.1 mg L^−1^ at 90 min, with low energy consumption of 7.1 ± 0.003 Wh g^−1^ and stable performance during 10 cycles of operation. Our results showed that this 3-D FAC is a promising approach for *in situ* H_2_O_2_ production for both environmental remediation and industrial applications.

## Introduction

Hydrogen peroxide (H_2_O_2_), as a green, powerful and versatile oxidant, has been widely applied either alone or as a reagent of advanced oxidation processes (e.g., Fenton/Fenton-like reactions^1,2^, TiO_2_/H_2_O_2_/UV photocatalysis^3,4^, ozone treatment^2^) for water/wastewater treatment, disinfectant and paper-blenching applications^2,5,6^. At present, H_2_O_2_ is commercially produced using the anthraquinone process in large-scale facilities, involving the sequential hydrogenation and oxidation of anthraquinone molecules^7,8^. This process is inherently complex and energy-intensive (1–2 dollars per kilogram), in which anthraquinone and its derivative are carcinogenic compounds^9^. Although H_2_O_2_ is not considered an explosive, the transportation, storage and handling of concentrated solutions need special safety precautions^10,11^. Therefore, many researchers now focus on the *in situ*, continuous and cleaner production of H_2_O_2_, primarily via the two-electron oxygen reduction reaction (ORR, O_2_ + 2H^+^ + 2e^−^ → H_2_O_2_)^12,13^.

Due to the limit of low solubility (8.1–8.5 ppm at 25 °C)^14,15^ and small diffusion coefficient (*D*_O2,water_ of 1.96–2.56 × 10^−9^ m^2^ s^−1^ at 25 °C)^16,17^ of oxygen in water, oxygen mass transfer has been recognized as an important rate-limiting step of oxygen reduction reaction (ORR)^18,19^. The air cathode, in which a hydrophobic gas diffusion layer (GDL) directly exposes to air (*D*_O2, GDL_ of 3.0 × 10^−6^ m^2^ s^−1^) and thus oxygen passively diffuses to its catalyst layer (CL, *D*_O2, CL_ 3.0 × 10^−7^ m^2^ s^−1^)^20^, no longer depends on the feed of dissolved oxygen in the electrolyte solution. The binders of GDL and CL are often superhydrophobic polymers, such as poly(dimethylsiloxane)^21^ and poly(tetrafluoroethylene) (PTFE)^22^, maintaining the O_2_ diffusion path and preventing catalyst flooding. Many reactors based on air cathode, such as stacked electrosynthesis reactor^23^ and divided-cell trickle bed electrochemical reactor^24^, were designed and applied for H_2_O_2_ production. When air cathodes are scaled up for practical applications, it is difficult to achieve uniform water infiltration due to both changing water pressures at different depth and immature GDL fabrication at a large scale^25,26^. This uneven water infiltration resulted in either water leakage, or a non-uniform current distribution within the cathode, leading to degraded cathode performance^25,26^.

The electrochemical ORR systems often rely on single external O_2_ supply methods such as passive oxygen diffusion or active aeration, but neglect the produced O_2_ from the anodic oxygen evolution reaction (OER)^10,19,27^. In the two-electron oxygen reduction process, the produced O_2_ from the OER anode is half of that consumed at the cathode^10,28^, but this part of O_2_ is often wasted. Recently, with the utilization of anodically produced oxygen, we developed an oxygen-self-supplied electro-fenton system with dual cathodes that did not need the feed of external O_2_ supply^10^. Similar approaches that utilize the anodically produced oxygen extend the application field of *in situ* electrochemical H_2_O_2_ production^3,29^. In spite of much work on single oxygen supply^18,30^, the electrochemical system to produce H_2_O_2_ with multi-oxygen supplies is rarely reported. In addition, it is really significant to figure out contributions of different O_2_ supplies to H_2_O_2_ production for the future practical applications.

In this study, in order to address the uneven water infiltration issues and enhance the O_2_ mass transfer to air cathodes for scale up applications, we developed a floating air cathode (FAC) using the commercially available poly(urethane) (PU) sponge that floats at the solution/air interface for effective H_2_O_2_ production without the needs of extensive water-proof measures. The sponge was dipped in the catalyst ink made of carbon black to make it electrically conductive and electrochemically active. The FAC with various dipping times (DTs) was characterized in terms of morphology, mass loading, ohmic resistance (*R*_ohm_), and electrochemically active surface area (ECSA). The FAC performance in terms of H_2_O_2_ concentration and normalized energy consumption were also evaluated to find the most cost-effective catalyst loading for the production of H_2_O_2_. We examined the H_2_O_2_ production with different relative positions of the cathode in the electrolyte, to characterize the influence of O_2_ supply sources (passive diffusion from air, and O_2_ produced from the OER anode) to the ORR system.

## Methods

### Electrode Preparation

**Figure 1:**
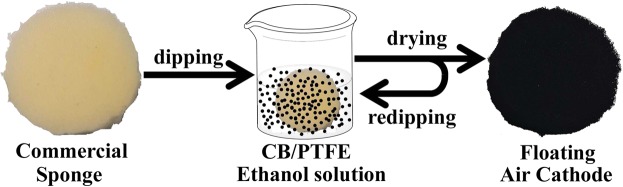
Schematic illustration of floating air cathode via the dipping-drying process.

The floating air cathode (FAC) was fabricated through a simple and scalable dipping and drying process (Fig. 1)^31^, around the commercially available PU sponge of 50 pores per inch (ppi) as the support (Hangmei sponge Co., Ltd). Carbon black (CB, acetylene, 50% compressed, Alfra Aesar Co., Ltd) was used both as the catalyst and the conductive layer, with PTFE (60 wt.% dispersion in H_2_O, Sigma-aldrich Co., Ltd) as the binder. CB (300 mg) and 60% PTFE (2 mL) with PTFE/CB ratio of 6:1 were dispersed in 30 mL of ethanol with ultrasonication of 30 min to form a uniform suspension. The sponge was dipped into the suspension and dried in the electric oven at 80 °C for several times^31^. The FAC had a diameter of 4.0 centimeters (cm, projected surface area of 12.56 cm^2^, 1.0 cm in height), and catalyst loading depended on the dipping times. Mixed metal oxides (MMO) mesh (4.0 cm in diameter × 0.1 cm height) was used as the anode.

### Operation

All the electrochemical measurements were performed in Petri dishes (6.0 cm inner diameter × 3.0 cm height or 9.0 cm inner diameter × 2.0 cm height, Fig. S1) with 50 mL electrolyte (100 mM Na_2_SO_4_ solution). The FAC floated at the solution/air interface, and the MMO anode was placed at the bottom of the reactor and facing the FAC. The electrode spacing between the bottom of FAC and the top of MMO was ~2.0 cm. The electrochemical double-layer capacitance (EDLC) measurements of FACs with different dipping times were carried out using cyclic voltammetry between −0.04 and 0.04 V versus Ag/AgCl with the scan rate of 0.5 mV s^−1^, which served as an estimate of the electrochemically active surface area (ECSA) of the solid-liquid interface^32,33^. FACs with different dipping times were measured by linear sweep voltammetry (LSV) from 0 to −1.0 V versus Ag/AgCl with the scan rate of 5 mV s^−1^ to evaluate their ORR performance. The production of H_2_O_2_ was measured at a fixed voltage of 2 V using the potentiostat (VMP3, BioLogic, France). Cathode potentials were measured by the Keithley data acquisition system (2700 multimeter, Keithley, America) versus the Ag/AgCl reference electrodes (all cathode potentials were reported versus Ag/AgCl). Under the same mode with the fixed voltage of 2 V, the FAC of DT6 was chosen to evaluate the stability of H_2_O_2_ generation by changing the electrolyte every 90 min for 10 times. The produced H_2_O_2_ concentrations were measured with different applied voltages using the FAC of DT6.

To investigate the influence of oxygen supply on system performance, four types of working modes were designed (Fig. S1), including: the mode as mentioned above with the floating air cathode facing the OER anode (Mode 1, M1, oxygen from both air and OER), floating air cathode misplaced with OER anode (Mode 2, M2, oxygen only from air), submerged cathode facing the OER anode (Mode 3, M3, oxygen from OER) and submerged cathode misplaced with OER anode (Mode 4, M4, no continuous oxygen supply). The produced H_2_O_2_ concentrations under different working modes were measured, under the same set voltage of 2 V.

### Analysis and Calculations

Scanning electron microscopy (SEM) images of FACs were taken on a JEOL JSM7001 scanning electron microscope with the accelerating voltage of 20 kV. The ohmic resistance was monitored by the digital multimeter (DM-A, Jetech Co. Ltd., China), and mass loading of the catalyst was calculated based on the weight difference measured by electronic balance (ME-104, Mettler Toledo, China).

The concentration of H_2_O_2_ standard solution was quantified by a classical potassium permanganate titration based on the below reaction (5H_2_O_2_ + 2MnO_4_^−^(red) + 6H^+^ → 2Mn^2+^(colorless) + 5O_2_ + 8H_2_O). The calculation of H_2_O_2_ concentration (*c*, g L^−1^) was

$$c({H}_{2}{O}_{2})=\frac{c(KMn{O}_{4})\times V(KMn{O}_{4})\times M({H}_{2}{O}_{2})\times 5}{M(KMn{O}_{4})\times V({H}_{2}{O}_{2})\times 2}$$

where *c*(KMnO_4_) was the concentration of KMnO_4_ standard solution (g L^−1^), *V*(KMnO_4_) the volume of consumed KMnO_4_ standard solution (L), *M*(KMnO_4_) the molar mass of KMnO_4_ (158 g mol^−1^), *M*(H_2_O_2_) the molar mass of H_2_O_2_ (34 g mol^−1^) and *V*(H_2_O_2_) the volume of titrated H_2_O_2_ standard solution (L).

The H_2_O_2_ concentration of sample was measured by titanium potassium oxalate method^10,19^ with a UV-vis spectrophotometer (UV-6850, Jenway, Britain). In the titanium potassium oxalate method, 200 mg L^−1^ of H_2_O_2_ standard solution was prepared by the as-titrated H_2_O_2_ solution. To obtain the calibration curve, 200 mg L^−1^ H_2_O_2_ standard solutions of 0.1 mL, 0.2 mL, 0.3 mL, 0.4 mL and 0.5 mL were added into 0.5 mL K_2_TiO(C_2_O_4_)_2_ (25 mmol L^−1^) in H_2_SO_4_ (1 mol L^−1^) solution, and then diluted with deionized water to 1 mL, and finally measured by a UV-vis spectrophotometer at the wavelength of 400 nm. Based on the linear relationship between the signal intensity and H_2_O_2_ concentration, the H_2_O_2_ concentrations of samples could be obtained. 0.5 mL of solution in each sample was collected for the quantification of H_2_O_2_ and then 0.5 mL of initial blank solution was added into the electrochemical system to maintain the volume of electrolyte.

The calculation of energy consumption (EC, Wh g^−1^)^22^ was

$${\rm{EC}}=\frac{E\times \int I\,dt}{3600\times c({{\rm{H}}}_{2}{{\rm{O}}}_{2})\times V}$$

where *I* was the ORR current (A), *E* the fixed voltage of 2 V, *t* the operation time (s), *V* the volume of electrolyte (L) and *c*(H_2_O_2_) the concentration of produced H_2_O_2_ (g L^−1^).

The calculation of coulombic efficiency (CE, %)^34^ was

$${\rm{CE}}=\frac{n\times F\times c({{\rm{H}}}_{2}{{\rm{O}}}_{2})\times V}{M({{\rm{H}}}_{2}{{\rm{O}}}_{2})\times \int I\,dt}\times 100 \% $$

where *I* was the ORR current (A), *n* the number of transferred electrons (*n* = 2), *F* the Faraday’s constant (*F* = 96485 C mol^−1^), *t* the operation time (s), *V* the volume of electrolyte (L), *M*(H_2_O_2_) the molar mass of H_2_O_2_ (34 g mol^−1^) and *c*(H_2_O_2_) the concentration of produced H_2_O_2_ (g L^−1^).

## Results and Discussion

### Characterization of the Floating Air Cathode

The three-dimensional PU sponge that has been commonly used for household cleaning, packaging, filtrating and many other applications, was utilized for the fabrication of the air cathode. The sponge was dipped into a CB/PTFE ethanol solution, allowing the solution to fill the voids and coat the skeletons. The as-formed electrode could freely float on the air/water interface while pumping water in or out the Petri dish, successfully maintaining the O_2_ diffusion path from air. Densities of the PU sponge before and after the dipping-drying treatment (0.02 g cm^−3^ for sponge, 0.05–0.12 g cm^−3^ for FAC, Table S1) were far less than that of water, guaranteeing the floating property of sponge-based electrodes.

**Figure 2:**
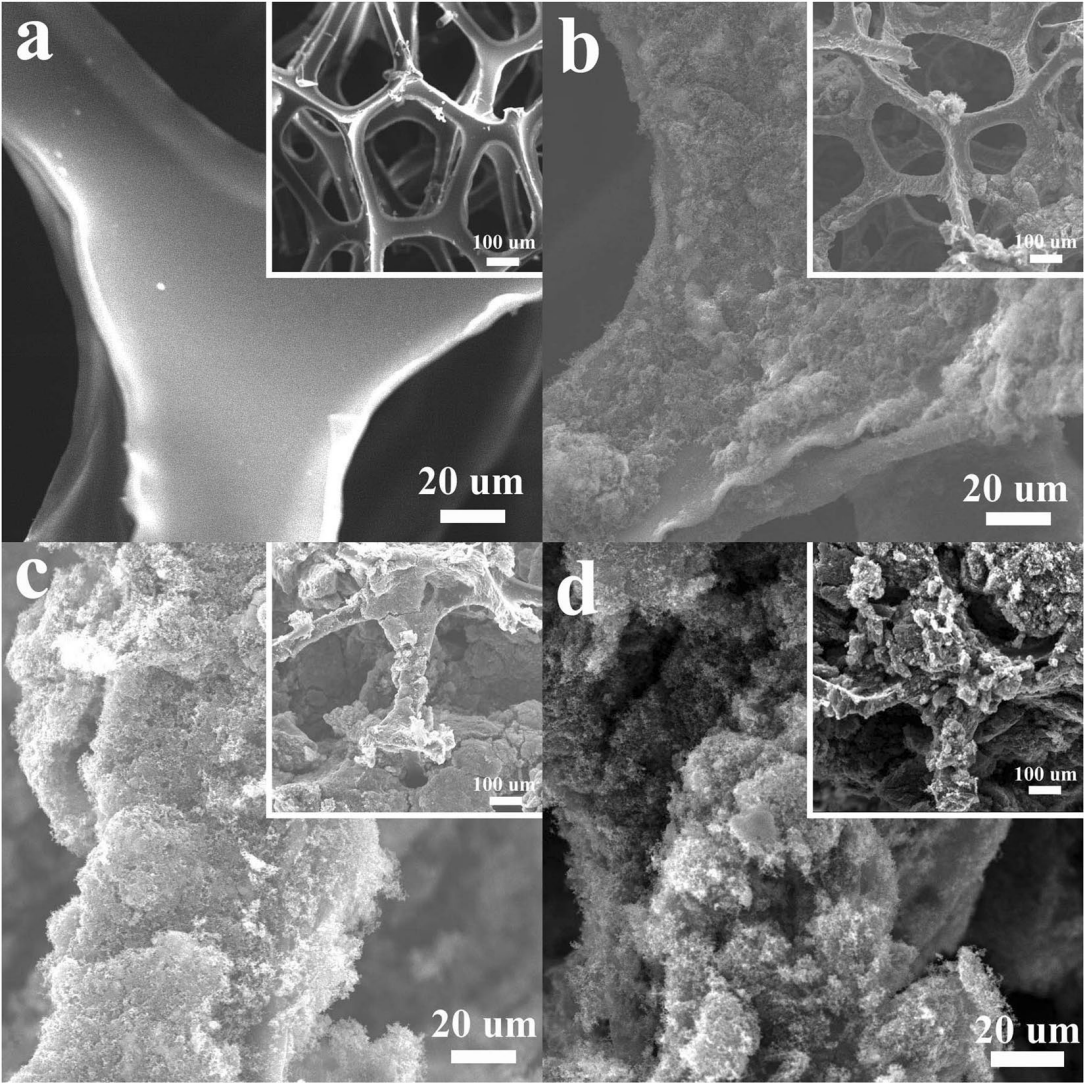
High resolution SEM images of (**a**) commercial sponge and floating air cathodes with different dipping-drying times, including (**b**) once, (**c**) three times and (**d**) six times. The insets are low resolution SEM images.

**Figure 3:**
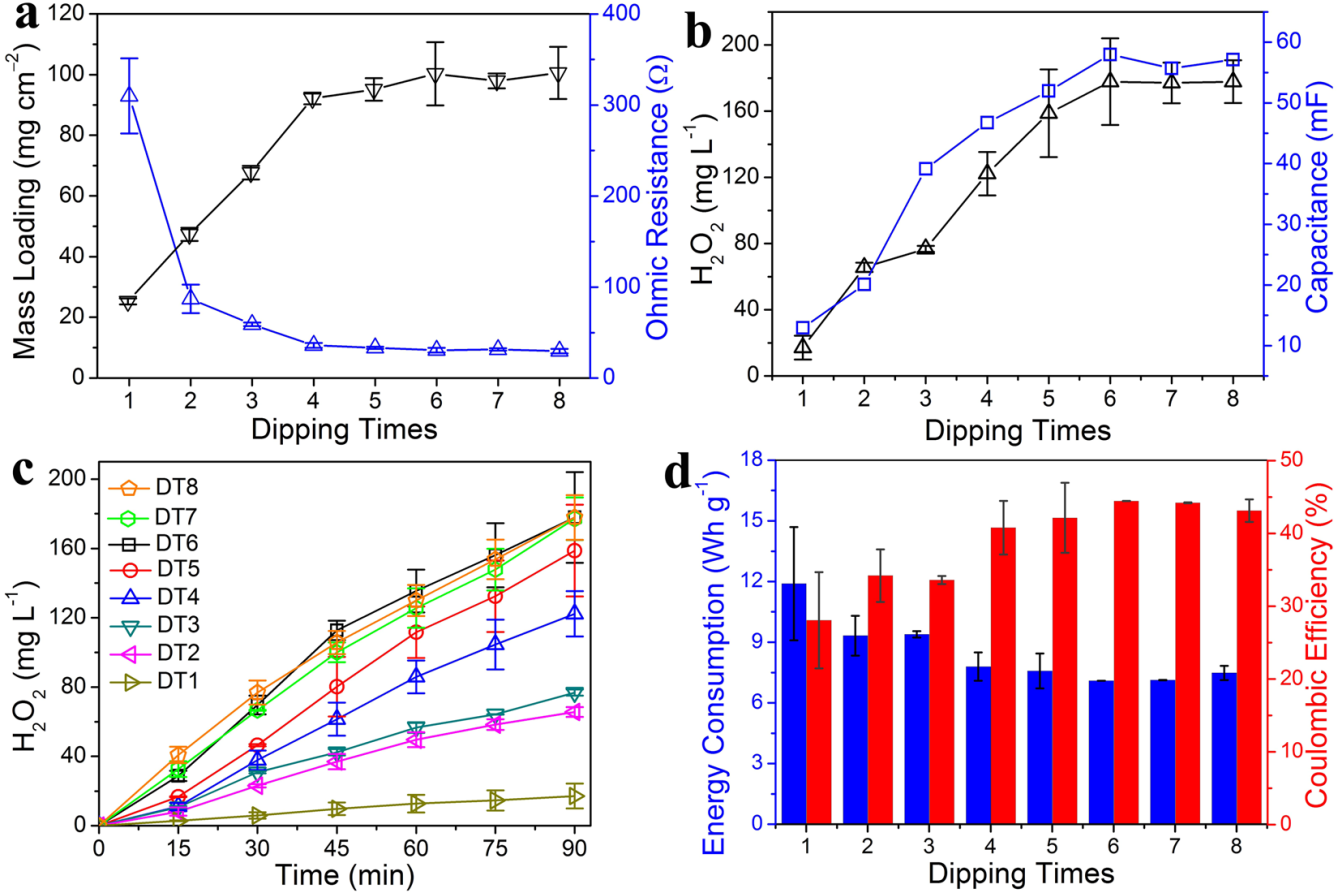
(**a**) Mass loading and ohmic resistances, (**b**) H_2_O_2_ production at 90 min and capacitance of electrochemical double layer, (**c**) H_2_O_2_ production with different reaction time, and (**d**) energy consumption and coulombic efficiency at 90 min of floating air cathodes with different dipping times.

The small size of CB powder and strong adhesion of PTFE binder enabled the formation of a carbon “skin” that coated on the sponge surface, as compared with SEM images of the sponge before and after the dipping-drying treatment (Fig. 2 and S2). With the increase of dipping times, the CB/PTFE layer became thicker and gradually filled macropores of the sponge (Figs 2 and S2). This CB/PTFE coating changed the color of sponge from yellow to black (Fig. 1) and electrical conductivity of the entire matrix from insulative to conductive (Fig. S3). In the FAC, the mass loading of catalyst and ohmic resistance greatly relied on dipping times. With the increase of DTs from one to four, the mass loading of catalyst hugely increased from 25.2 to 92.1 mg cm^−2^ while ohmic resistance rapidly decreased from 310 Ω to 36 Ω (Fig. 3a). After four times of dipping into the CB/PTFE solution, the mass loading of catalyst and ohmic resistance of FACs tended to be stable. With six times of dipping-drying treatment, the ohmic resistance of PU sponge coated with CB/PTFE mixture was ~31 Ω (Figs 3a and S3b), enabling its function as an electrode in small scale applications.

### Effect of dipping times on FAC electrochemical performance

To estimate the electrochemically active surface area (ECSA) of FACs with different dipping times, the electrochemical double-layer capacitance (EDLC) were measured by cyclic voltammetry^35,36^. It showed that the capacitance greatly relied on dipping times of the electrode. With the increasing DTs from one to six, the EDLC of FACs gradually increased from 12.9 to 58.0 mF (Figs 3b and S4), suggesting that the ECSA improved due to the increased catalyst mass loading. Although the measured resistance did not appreciablely decrease after four times, the ECSA still improved with a higher catalyst loading. After six times of dipping treatment, the EDLCs of FACs started to be stable (55.7 mF of DT7 and 57.1 mF of DT8, Figs 3b and S4), attributing to their similar mass loading and ohmic resistance to DT6 (97.9 mg cm^−2^ and 32 Ω for DT7, 100.5 mg cm^−2^ and 30 Ω for DT8, Fig. 3a).

To evaluate the ORR performance of FACs with various dipping times, linear sweep voltammetry was measured. Due to the same catalytic component, the onset potentials of all FACs were −0.27 V vs. Ag/AgCl (Fig. S5). However, the ORR performance of FACs improved with dipping times. At −1.0 V vs. Ag/AgCl, only −4.6 mA of ORR current was produced on the DT1 FAC, much less than −18.1 mA for DT2. Raising dipping times resulted in increasing ORR current from −31.2 mA for DT3 to −42.5 mA for DT4 and −51.2 mA for DT5. When dipping time was eight, the ORR current reached a maximum of −63.2 mA, slightly larger than those for DT6 (−61.4 mA) and DT7 (−58.3 mA, Fig. S5). The improved ORR performance with DTs was attributed to higher active surface area and lower resistance that resulted from the increased catalyst mass loading. This was consistent with previous studies that mass loading of catalyst, ECSA and electrical conductivity of electrode were positively correlated with the performance of electrochemical systems^34,37^. Thus, when the same set voltage of 2 V was applied to produce H_2_O_2_, cathode potentials became more positive for the FACs with more DTs (Fig. S6). On average, the cathode potential on DT6 FAC was −0.63 V, a little more positive than −0.68 V on DT5 and −0.71 V on DT4. When dipping times were two or three, the average cathode potential was close, approximately −0.75 V (DT3) and −0.78 V (DT2). The FAC that was dipped only once had the most negative cathode potential of −0.88 V (Fig. S6), suggesting the worst cathode performance.

Produced H_2_O_2_ were measured using FACs with different dipping times. With the fixed set voltage of 2 V, the cathodic current and H_2_O_2_ production showed a positive relationship with the DTs of FACs from once to six times (Figs 3c and S7), attributing to the increasing mass loading, decreasing ohmic resistance and increasing ECSA. When the dipping times were six, the H_2_O_2_ concentration reached a maximum of 177.9 ± 26.1 mg L^−1^ at 90 min, slightly more than 158.7 ± 26.5 mg L^−1^ for five times (Fig. 3c). Lowering the DTs of FAC led to decreased H_2_O_2_ concentration from 122.2 ± 13.1 mg L^−1^ for four times to 65.6 ± 2.8 mg L^−1^ for twice (Fig. 3c). Only 17.2 ± 7.2 mg L^−1^ H_2_O_2_ generated if the sponge was dipped into CB/PTFE solution only once (Fig. 3c). The improved H_2_O_2_ concentration with DTs from once to six times resulted from larger cathodic current (Fig. S7) and higher coulombic efficiency (Fig. 3d). However, after six times of dipping, the FACs showed similar electrochemical performance, leading to stable H_2_O_2_ generation of 177.1 ± 12.2 mg L^−1^ for seven times and 177.8 ± 12.9 mg L^−1^ for eight times (Fig. 3c).

Normalized energy consumption was evaluated for the H_2_O_2_ production using the FACs. The lowest consumed energy of 7.1 ± 0.003 Wh g^−1^ was obtained at six dipping times for the FACs, close to 7.1 ± 0.01 Wh g^−1^ at seven times, 7.5 ± 0.35 Wh g^−1^ at eight times (Fig. 3d). With the decreased dipping times, energy consumption gradually increased from 7.6 ± 0.9 (five times) to 11.9 ± 2.8 (once) Wh g^−1^ due to the increased ohmic resistance. The FAC was further evaluated by comparing its energy consumption with those of CB-based gas diffusion electrodes (GDEs) reported previously (Table 1)^13,22,27,38–41^. The energy consumption obtained here were lower than those with conventional CB-based GDEs (7.45–22.1 Wh g^−1^, Table 1)^13,22,27,40^. For the modified cathodes with CoPc or FePc addition, the energy consumption was higher to be 30.8–165 Wh g^−1^ ^39,41^. The cathode with tert-butyl-anthraquinone (TBAQ) addition showed superior performance (6.0 Wh g^−1^)^38^ due to improved two-electron transfer selection, indicating that the energy consumption in our system could be further lowered with the improvement of catalyst activity.

**Table 1:** The comparisons of energy consumption of H_2_O_2_ production and H_2_O_2_ generating rate using the floating air cathode and other gas diffusion electrodes.

Electrode	Electrode Orientation and Area	Catalyst	Volume and Type of Electrolyte	Electrochemical Method	H_2_O_2_ Concentration@Time	H_2_O_2_ Generating Rate (mg h^−1^ cm^−2^)	Energy Consumption (Wh g^−1^)	ref.
Floating Air Cathode	Horizon, 12.56 cm^2^	Carbnon Black (CB)	50 mL, 100 mM Na_2_SO_4_	a fixed voltage of 2 V	177.9 ± 26.1 mg L^−1^, 90 min	0.46–0.60	5.9–7.1	This work
Floating Air Cathode	Horizon, 12.56 cm^2^	CB	50 mL, 100 mM Na_2_SO_4_	a fixed voltage of 5 V	1062.1 ± 79.4 mg L^−1^, 90 min	2.82–5.80	11.1–23.8	This work
Gas Diffusion Cathode	Vertical, 20 cm^2^	CB, tert-butyl-anthraquinone	400 mL, 100 mM K_2_SO_4_, 100 mM H_2_SO_4_	a fixed potential of −1.1 V vs. SCE	301 mg L^−1^, 90 min	4.01	6.0	^38^
Gas Diffusion Cathode	Vertical, 100 cm^2^	CB	1 L, 1 g L^−1^ Na_2_SO_4_ + H_2_SO_4_, pH = 3	a fixed current of 3 A	1000 mg L^−1^, 35 min	17.14	7.45	^27^
Gas Diffusion Cathode	Vertical, 20 cm^2^	CB	400 mL, 1000 mM KOH	a fixed potential of −1.1 V vs. Ag/AgCl	3370 mg L^−1^, 90 min	44.93	8.0	^13^
Gas Diffusion Cathode	Vertical, 14.13 cm^2^	CB	200 mL, 50 mM Na_2_SO_4_	a fixed current of 100 mA	556 mg L^−1^, 180 min	2.62	8.6	^22^
Gas Diffusion Cathode	Vertical, 20 cm^2^	CB	1.5 L, 100 mM K_2_SO_4_, 100 mM H_2_SO_4_	a fixed potential of −2.25 V vs. Ag/AgCl	414 mg L^−1^, 120 min	15.53	22.1	^40^
Gas Diffusion Cathode	Vertical, 20 cm^2^	CB, Cobalt (II) phthalocyanine	400 mL, 100 mM K_2_SO_4_, 100 mM H_2_SO_4_	a fixed potential of −0.7 V vs. Ag/AgCl	331 mg L^−1^, 90 min	4.41	30.8	^39^
Gas Diffusion Cathode	Vertical, 20 cm^2^	CB, Iron (II) phthalocyanine	400 mL, 100 mM K_2_SO_4_, 100 mM H_2_SO_4_	a fixed potential of −1.0 V vs. Ag/AgCl	240 mg L^−1^, 90 min	3.2	165	^41^

### Effect of applied voltages on H_2_O_2_ generation

**Figure 4:**
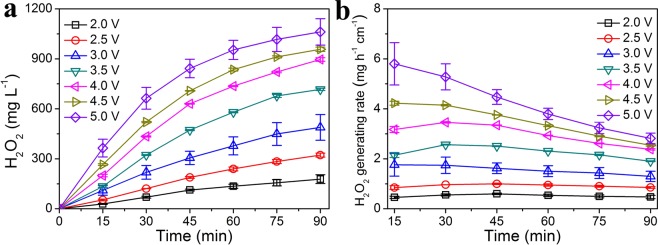
(**a**) H_2_O_2_ production and (**b**) H_2_O_2_ generating rate with different reaction time of floating air cathodes at different applied voltages.

H_2_O_2_ production was measured when applying different voltages using the FAC of DT6. With the set voltage increasing from 2.0 V to 5.0 V, cathodic current gradually increased from 25.6 mA to 170.9 mA (Fig. S8), suggesting that oxygen reduction on the FAC dramatically enhanced. This huge ORR enhancement led to the improving performance of H_2_O_2_ generation via two-electron ORR pathway. As a result, the increase of H_2_O_2_ concentration with applied voltage was from 177.9 ± 26.1 mg L^−1^ at 2.0 V to 488.6 ± 77.5 mg L^−1^ at 3.0 V and 896.4 ± 10.3 mg L^−1^ at 4.0 V (Fig. 4a). At the set voltage of 5.0 V, the DT6 FAC produced a maximum of 1062.1 ± 79.4 mg L^−1^ H_2_O_2_ within 90 min (Fig. 4a).

Normalized H_2_O_2_ generating rate was calculated to evaluate the performance of FAC-based electro-generation system. H_2_O_2_ generating rate showed a positive relationship with the applied voltage from 2.0 V to 5.0 V (Fig. 4b). At the fixed voltage of 2.0 V, H_2_O_2_ was generated at the rate of only 0.46–0.60 mg h^−1^ cm^−2^, far less than 1.30–1.76 mg h^−1^ cm^−2^ at 3.0 V and 2.38–3.46 mg h^−1^ cm^−2^ at 4.0 V (Fig. 4b). When 5.0 V of voltage was applied, the DT6 FAC produced H_2_O_2_ at a maximum rate of 2.82–5.80 mg h^−1^ cm^−2^ (Fig. 4b). However, within 90 min of electrochemical treatment, H_2_O_2_ generating rate gradually decreased from 5.80 ± 0.85 mg h^−1^ cm^−2^ at 15 min to 2.82 ± 0.21 mg h^−1^ cm^−2^ at 90 min (Fig. 4b), attributing to enhanced H_2_O_2_ electro-decomposition (H_2_O_2_ reduction at cathode^1,18,42,43^ and oxidation at anode^42,43^) from the rise of accumulated H_2_O_2_ concentration and electrode potential. The rate of 5.80 ± 0.85 mg h^−1^ cm^−2^ to generate H_2_O_2_ obtained by FAC was higher than many reported GDEs (2.62–4.41 mg h^−1^ cm^−2^, Table 1)^22,38,39,41^. In the electrochemical flow-by reactors, H_2_O_2_ generating rate could be improved to 15.53–17.41 mg h^−1^ cm^−2^ (Table 1)^27,40^ due to low solution resistance derived from the extremely small interelectrode gap (2.0–6.5 mm). In addition, concentrated alkaline solution as highly-conductive and high-pH electrolyte was beneficial for electro-reduction of oxygen, resulting in an ultrahigh H_2_O_2_ generating rate of 44.93 mg h^−1^ cm^−2^ (Table 1)^13^. Therefore, H_2_O_2_ generating rate of FAC could be further improved by decreasing solution resistance and increasing electrolyte pH.

### Effect of different O_2_ supplies and electrode relative positions on the cathode performance

**Figure 5:**
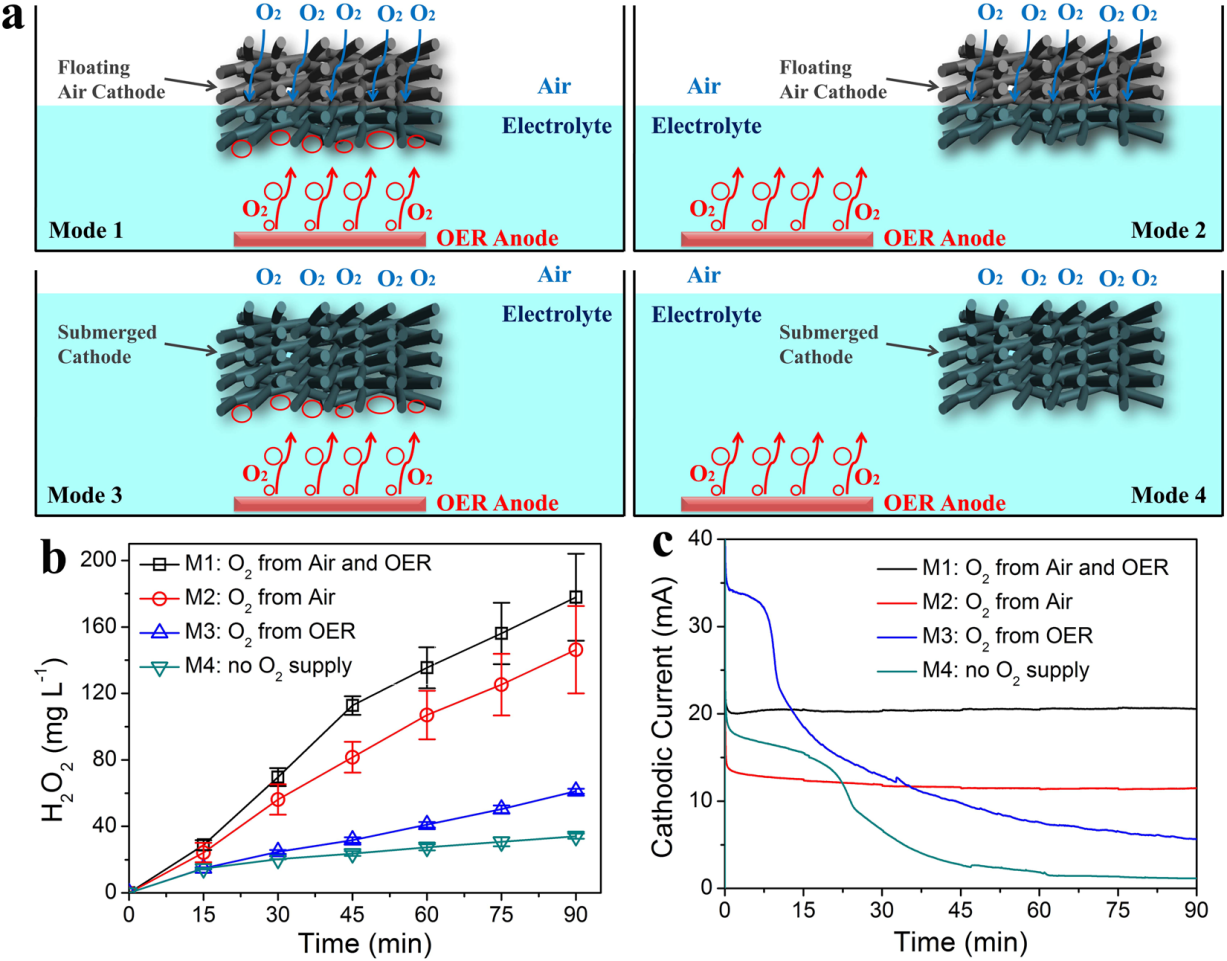
(**a**) Schematic illustrations of four working modes; (**b**) H_2_O_2_ production and (**c**) ORR cathodic current of the sponge-based electrodes under four working models with the set voltage of 2 V.

To investigate the effect of different O_2_ supplies on the cathode performance, H_2_O_2_ generation under four working modes was measured. These modes represented several different types of oxygen supply approach to the 3-D sponge-based electrode in the ORR system (Figs 5a and S1). The M1 working mode enabled continuous O_2_ supply of the FAC from air and anodic OER, resulting in the H_2_O_2_ production of 177.9 ± 26.1 mg L^−1^ within 90 min (Fig. 5b). The generated H_2_O_2_ concentration decreased to 146.3 ± 26.3 mg L^−1^ for M2 (O_2_ only from air) and 61.2 ± 1.4 mg L^−1^ for M3 (O_2_ only from anodic OER, Fig. 5b). The appreciable decrease in H_2_O_2_ concentration could be contributed to the increase of resistances (insert of Fig. 5c) and reduced O_2_ supply, indicating the significance of dual O_2_ supply for the energy-efficient H_2_O_2_ production using the FAC. Under the M4 condition with the submerged cathode and misplaced anode, the H_2_O_2_ generation of 34.1 ± 1.6 mg L^−1^ at 90 min (Fig. 5b) might be attributed to the little oxygen adsorbed in the sponge-based electrode and dissolved in the electrolyte (dissolved oxygen of 7.8–15.7 mg L^−1^, Fig. S9). Due to the lack of continuous oxygen supply, the cathodic current gradually decreased from 40 mA to 2 mA in the initial 45 min (Fig. 5c).

Compared with submerged cathodes (M3 and M4), the cathodic currents of FACs (M1 and M2) with O_2_ supply from air were relatively stable during the whole 90-min operation (Fig. 5c), indicating the steady O_2_ diffusion paths in FACs. The stable current production with FACs (M1 of 20.57 mA and M2 of 11.49 mA) were higher than those with submerged cathodes (M3 of 5.64 mA and M4 of 1.15 mA, Fig. 5c). The higher cathodic current with FACs resulted from passive O_2_ diffusion from air through the three-phase interface. When cathodes were submerged, the O_2_ transfer was greatly hindered in aqueous solution. Therefore, the H_2_O_2_ generation from FACs was also much better than that from submerged cathodes.

For the FACs (M1 and M2) and submerged cathodes (M3 and M4), the relative position between cathode and anode also affected their electrochemical performance. From a comparison of either M1&M2 or M3&M4, the cathodic current with a facing position was larger than those with a misplaced position (M1 of 20.6 mA > M2 of 11.5 mA and M3 of 5.6 mA > M4 of 1.2 mA, Fig. 5c). The decreased cathodic current with a misplaced position could be attributed to the increased solution resistance, electric field lines change and less oxygen supply without the utilization of OER produced oxygen. When the sponge-based cathodes were misplaced with MMO anodes, the solution resistance increased from 14.7 Ω for M1 to 27.7 Ω for M2 and from 7.4 Ω for M3 to16.2 Ω for M4 (Fig. S10). Therefore, consistent with the trend of cathodic current, the H_2_O_2_ production with a facing position was much higher than that with a misplaced position (Fig. 5b).

Coulombic efficiency was evaluated for the sponge electrodes under all four working modes. The CEs for FACs ranged from 39.7 ± 0.2% to 77.7 ± 4.4%, and the highest value of 77.7 ± 4.4% was obtained for M2 at 30 min (Fig. S11). However, for submerged cathodes, the CEs were lower than 30%, and the lowest CE of 15.9 ± 1.1% for M3 at 15 min resulted from the largest cathodic current and least H_2_O_2_ generation (Fig. S11). In general, the CEs for FACs (39.7 ± 0.2%–77.7 ± 4.4%) were also much higher than that for submerged cathodes (15.9 ± 1.1%–30.8 ± 2.7%, Fig. S11).

### Stability of the FAC for H_2_O_2_ production

**Figure 6:**
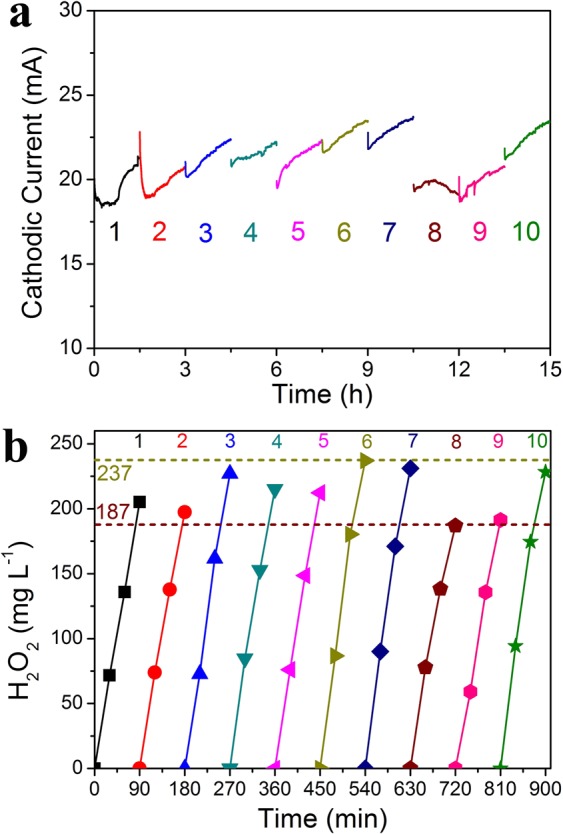
(**a**) ORR cathodic current and (**b**) H_2_O_2_ generation of ten cycles by changing the electrolyte every 90 min.

To evaluate the working stability of FAC with dual O_2_ supplies, the H_2_O_2_ generation with a fixed voltage of 2 V (the actual cathode potential ranged between −0.68 V and −0.56 V, Fig. S6) in a batch of experiments by changing the electrolyte every 90 min was investigated. Under the M1 working mode, the varying range of cathodic current on the FAC was moderate (18–24 mA, Fig. 6a), indicating the relatively stable performance of the FAC. In each run, the linear increase of H_2_O_2_ production also reflected the stability and sustainability of two-electron ORR process (Fig. 6b). The yield of H_2_O_2_ was almost stable with a fluctuation during ten cycles, and the fluctuating range of H_2_O_2_ concentration was from 187 to 237 mg L^−1^ (Fig. 6b). The fluctuation of H_2_O_2_ production could be partially resulted from the variation of ECSA of FAC in different cycles as the cathode floated freely on the solution/air interface. This result showed that the electrochemical ORR system using the FAC was capable of working stably, representing a promising system for energy efficient H_2_O_2_ production.

## Conclusions

In this study, the floating air cathodes were fabricated using commercially available sponge with a simple dipping-drying method. When the cathode floated on top of the OER anode, it had dual oxygen supply sources both from the air and anodic OER. The optimized FAC produced a maximum H_2_O_2_ production of 177.9 ± 26.1 mg L^−1^ within 90 min, and had a low energy consumption of 7.1 ± 0.003 Wh g^−1^ and good working stability. The features make the FAC a promising option to *in situ* H_2_O_2_ production in scale up applications for either environmental remediation or industrial applications.

## Supplementary information

Supporting Information
