# Supplementary material for: A Three-dimensional Floating Air Cathode with Dual Oxygen Supplies for Energy-efficient Production of Hydrogen Peroxide
Source: Sci Rep. 2019 Feb 12;9:1817. doi: 10.1038/s41598-018-37919-3 (PMC6372640; doi:10.1038/s41598-018-37919-3)
Supplement: Supplementary file 1 — Supporting Information [file 41598_2018_37919_MOESM1_ESM.docx]

- Supporting Information -

**A Three-dimensional Floating Air Cathode with Dual Oxygen Supplies for Energy-efficient Production of Hydrogen Peroxide**

Haichuan Zhang^1,2^, Yingjie Li^3^, Hao Zhang^1,2^, Guanghe Li^1,2^, and Fang Zhang^1,2,*^

^1^School of Environment and State Key Joint Laboratory of Environment Simulation and Pollution Control, Tsinghua University, Beijing, 100084

^2^Key Laboratory for Solid Waste Management and Environment Safety (Tsinghua University), Ministry of Education of China, Tsinghua University, Beijing 100084, China

^3^Department of Materials Science and Engineering, College of Engineering, Peking University, 100871, P. R. China

^*^Corresponding. [fangzhang@tsinghua.edu.cn](file:///F:\学习\文章\Floating%20Air%20Cathode\文章\SR\fangzhang@tsinghua.edu.cn), phone: 86-10-62789655

| Dipping Times | Weight 1 (g) | Weight 2 (g) | Weight 3 (g) | Average Weight (g) | Average Density (g cm^-3^)**^a^** |
| --- | --- | --- | --- | --- | --- |
| 0 | 0.3046 | 0.2955 | 0.2794 | 0.2932 | 0.0233 |
| 1 | 0.5842 | 0.6122 | 0.6338 | 0.6101 | 0.0486 |
| 2 | 0.8429 | 0.8968 | 0.8899 | 0.8765 | 0.0698 |
| 3 | 1.1199 | 1.1241 | 1.1828 | 1.1423 | 0.0909 |
| 4 | 1.4922 | 1.4376 | 1.4596 | 1.4631 | 0.1165 |
| 5 | 1.4524 | 1.5495 | 1.4614 | 1.4878 | 0.1185 |
| 6 | 1.3839 | 1.6014 | 1.6663 | 1.5505 | 0.1235 |
| 7 | 1.5571 | 1.4774 | 1.5084 | 1.5143 | 0.1206 |
| 8 | 1.4589 | 1.6001 | 1.6063 | 1.5550 | 0.1238 |

**Table S1.** Weight and density of floating air cathodes with different dipping times.

**a** The volume of floating air cathode (4.0 cm in diameter × 1.0 cm height) is calculated to 12.56 cm^3^.


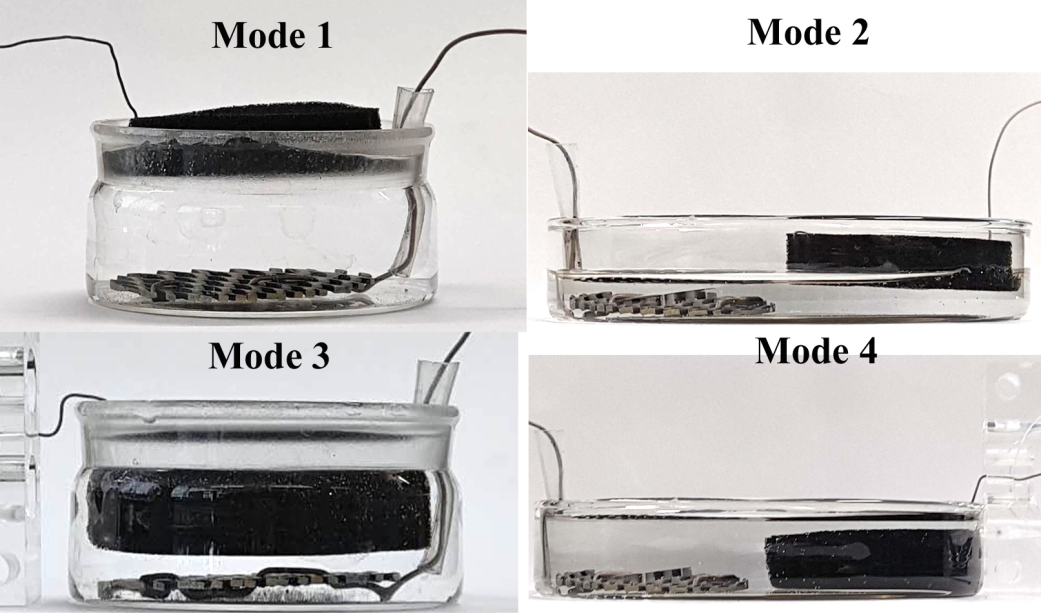


**Figure S1.** Optical images of four working modes.


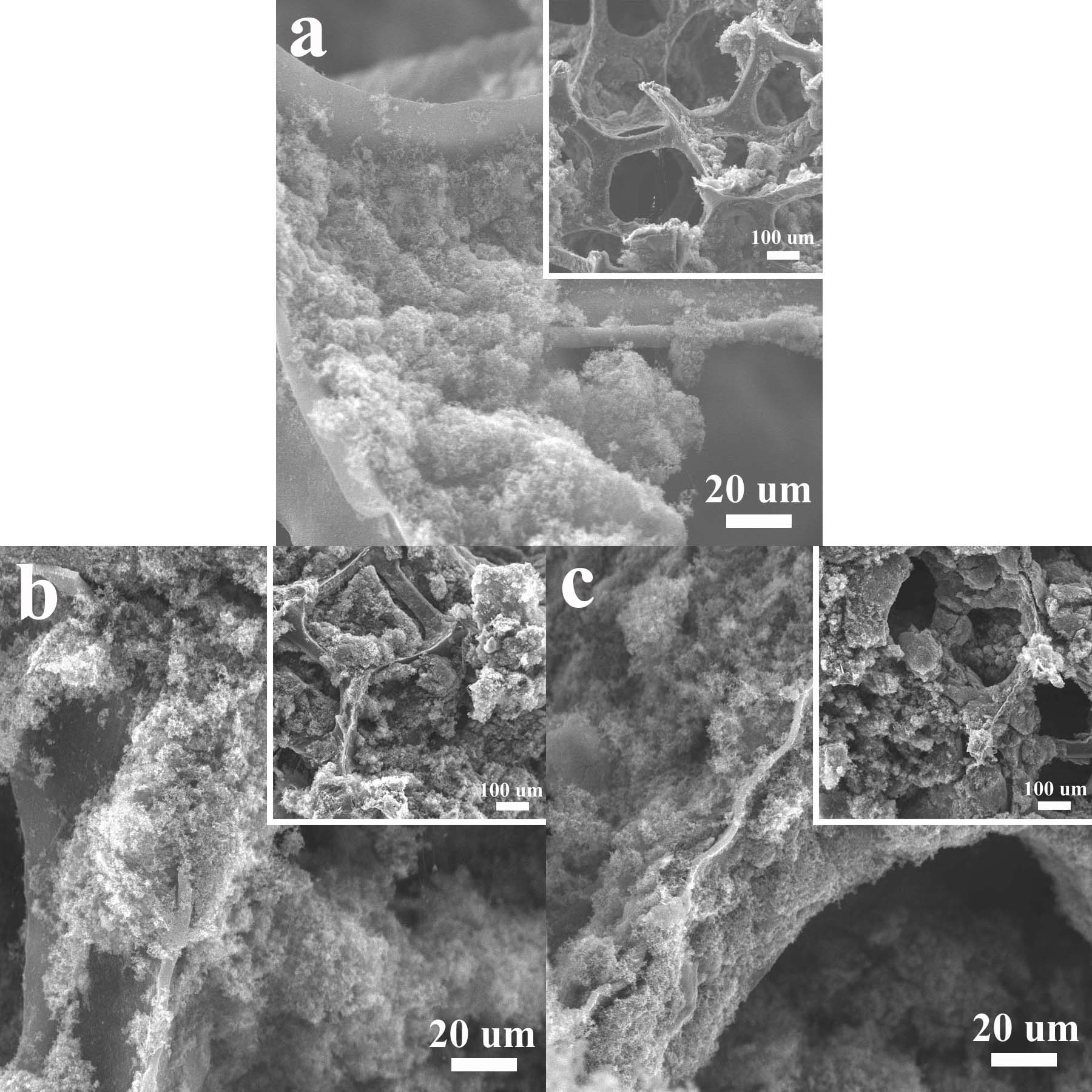


**Figure S2.** High resolution SEM images of floating air cathodes with different dipping-drying times, including (a) two times, (b) four times and (c) five times. The insets are low resolution SEM images.


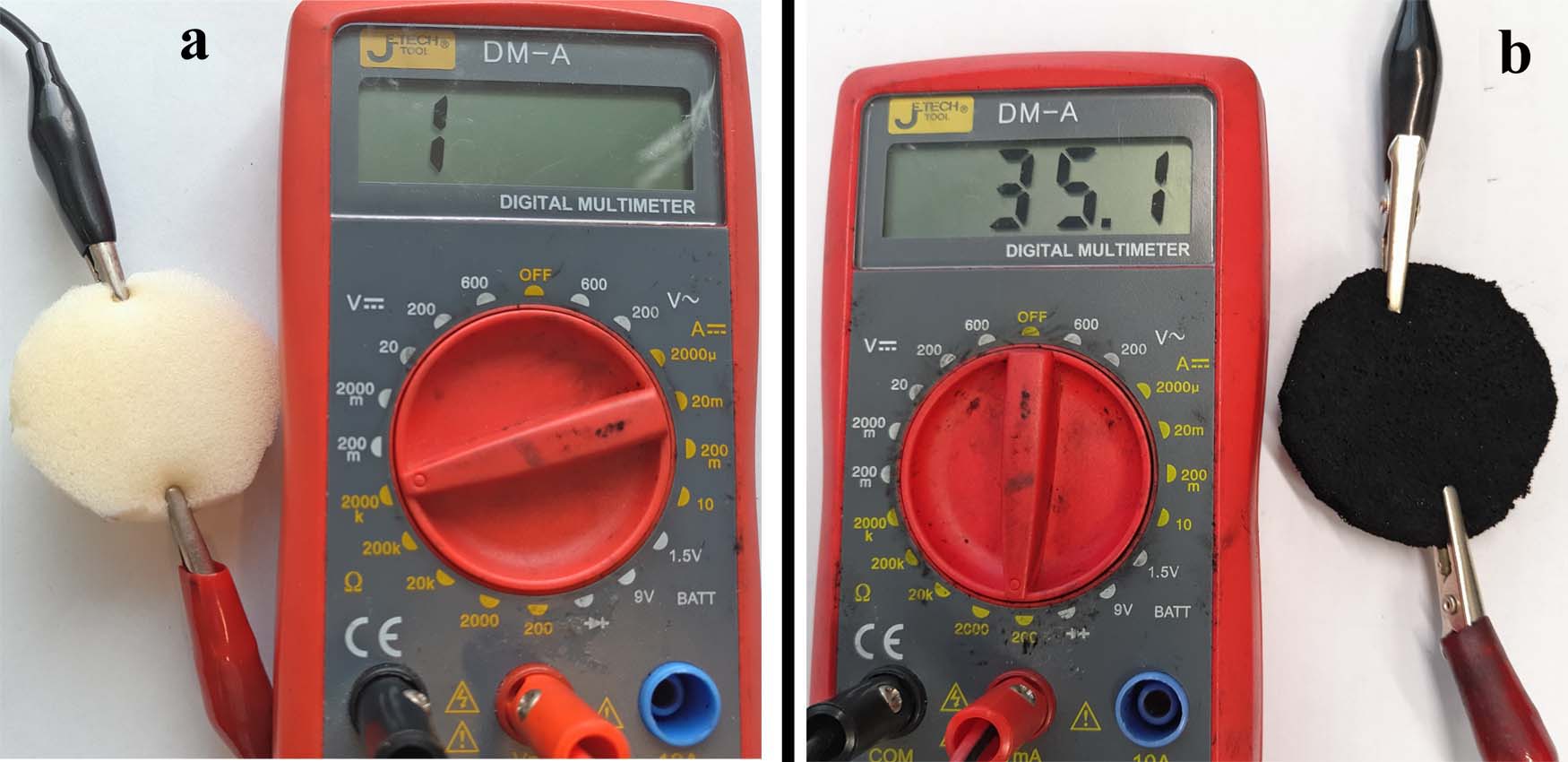


**Figure S3.** Ohmic resistances of (a) commercial sponge and (b) floating air cathodes with six dipping-drying times.


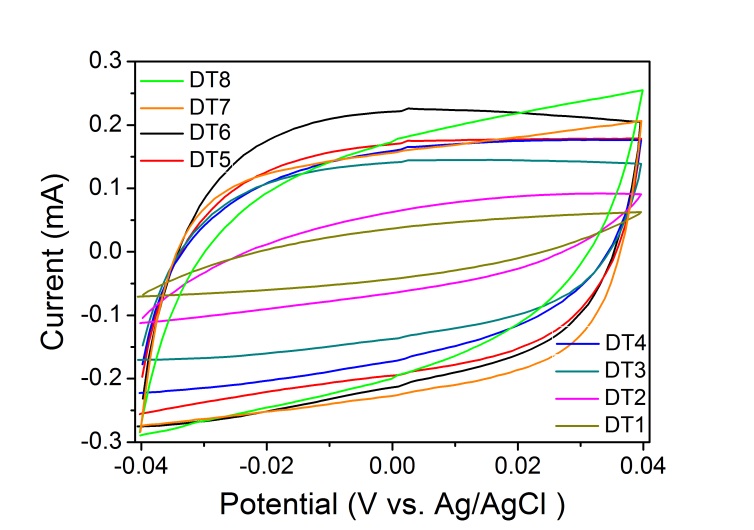


**Figure S4.** Cyclic voltammograms between -0.04 V and 0.04 V vs. Ag/AgCl of the floating air cathodes with different dipping times.


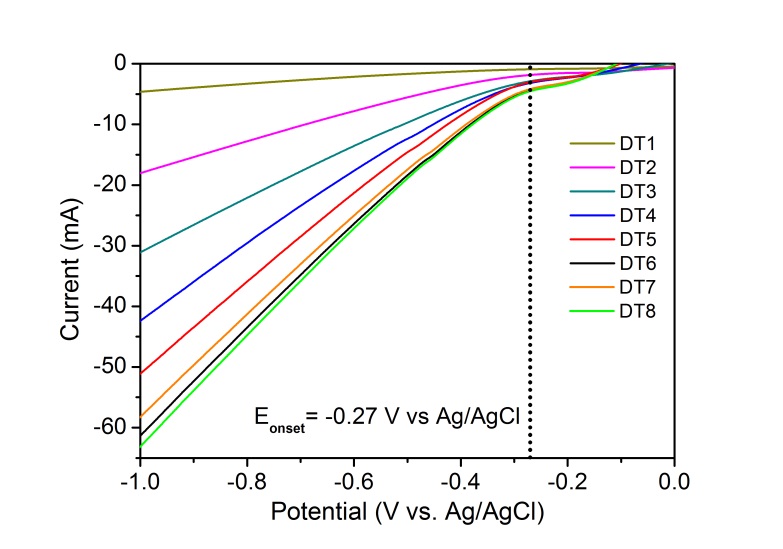


**Figure S5.** LSV curves of FACs with different dipping times from 0 to ‒1.0 V vs. Ag/AgCl.


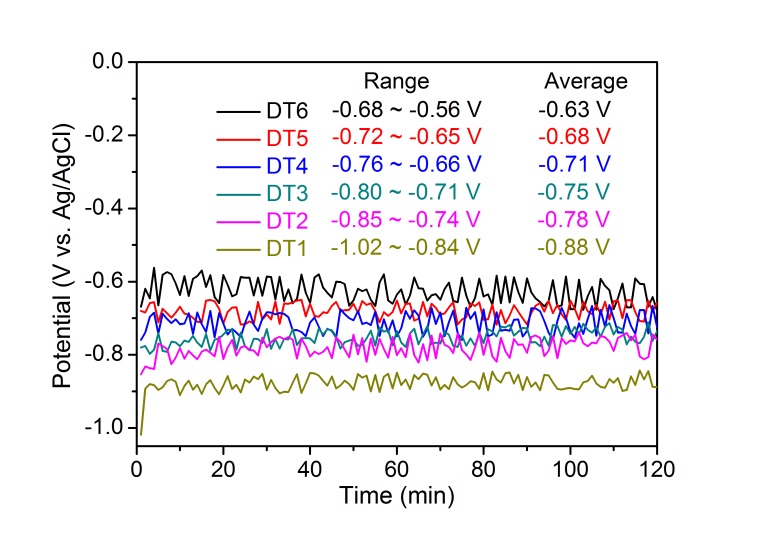


**Figure S6.** Cathode potential curves of FACs with different dipping times under the set voltage of 2 V.


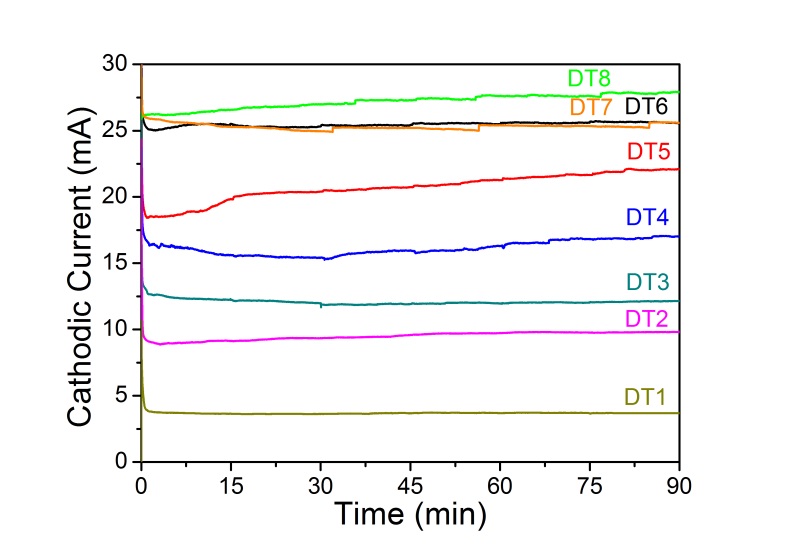


**Figure S7.** ORR cathodic current curves of FACs with different dipping times under the set voltage of 2 V.


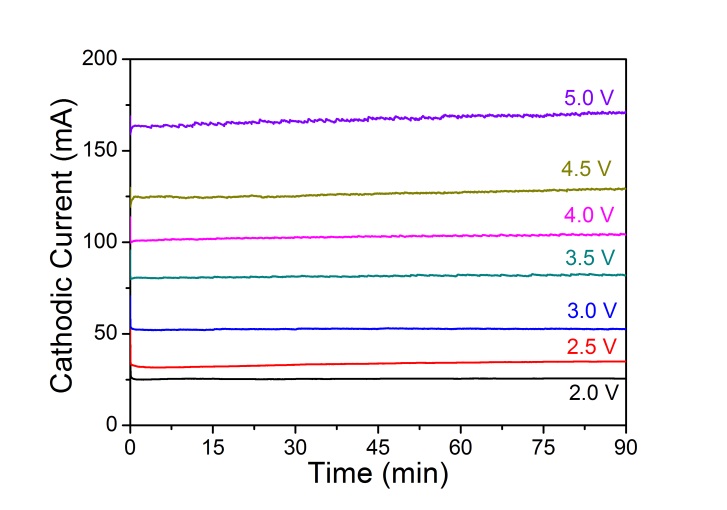


**Figure S8.** ORR cathodic current curves of the DT6 FAC under different applied voltages.


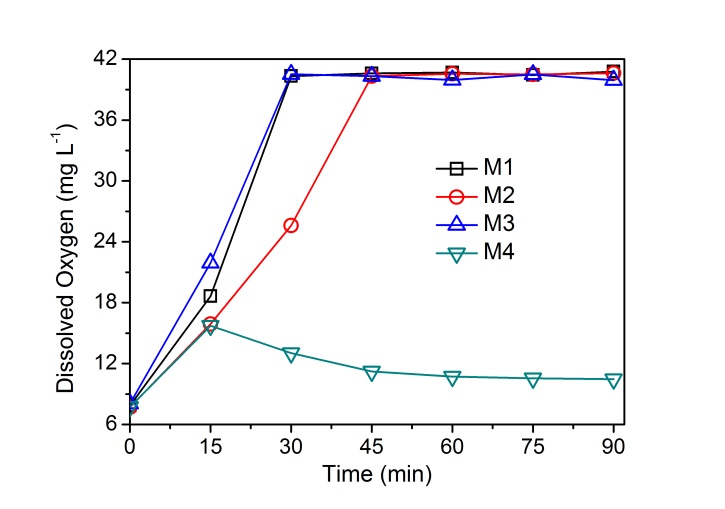


**Figure S9.** Dissolved oxygen concentrations with reaction time for the sponge-based electrodes at the set voltage of 2 V under four working modes.


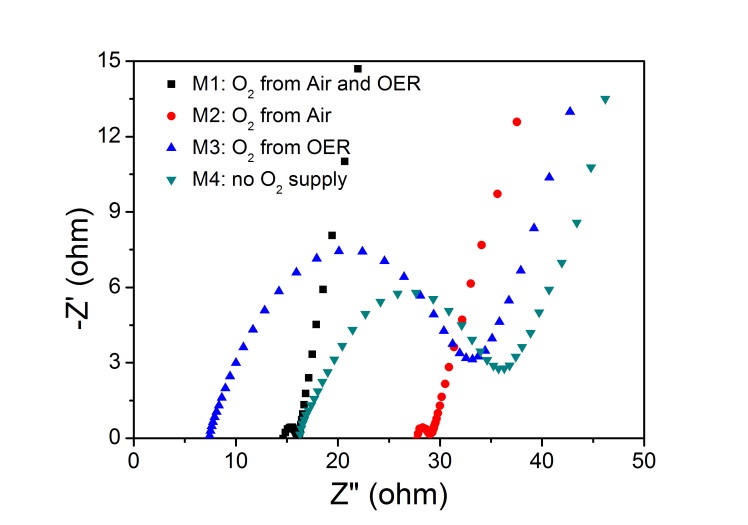


**Figure S10.** Nyquist plots of the sponge-based electrodes under four working modes.

*
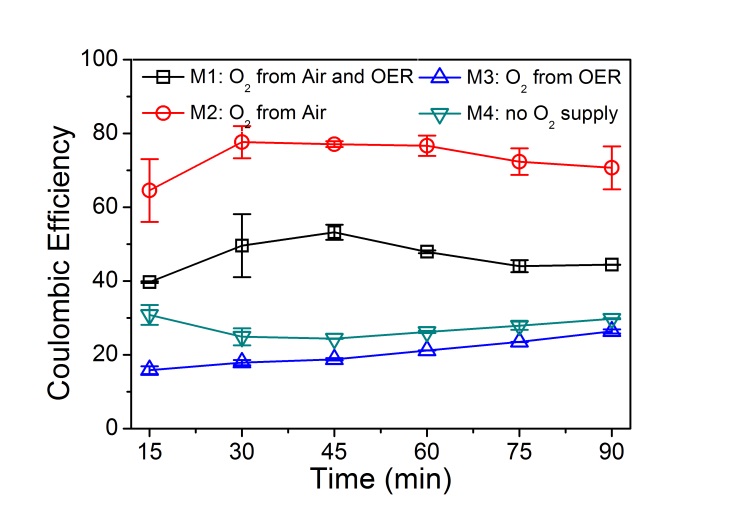
*

**Figure S11.** Coulombic efficiencies of the sponge-based electrodes under four working modes.
